# The Nutri-Score Scale—A Tool for Assessing the Nutritional Quality of Processed Meat Products Available on the Polish Market

**DOI:** 10.3390/nu16060827

**Published:** 2024-03-14

**Authors:** Katarzyna Czech-Załubska, Anna Didkowska, Daniel Klich, Agnieszka Jackowska-Tracz, Joanna Zarzyńska, Krzysztof Anusz

**Affiliations:** 1 Department of Food Hygiene and Public Health Protection, Institute of Veterinary Medicine, Warsaw University of Life Sciences (SGGW), Nowoursynowska 166, 02-787 Warsaw, Poland; katarzyna_czech_zalubska@sggw.edu.pl (K.C.-Z.); agnieszka_jackowska_tracz@sggw.edu.pl (A.J.-T.); joanna_zarzynska@sggw.edu.pl (J.Z.); krzysztof_anusz@sggw.edu.pl (K.A.); 2 Institute of Animal Sciences, Warsaw University of Life Sciences (SGGW), Ciszewskiego 8, 02-786 Warsaw, Poland; daniel_klich@sggw.edu.pl

**Keywords:** front-of-pack labelling system, Nutri-Score, processed meat products, Polish market

## Abstract

Although meat and meat products are important sources of protein in the human diet, consumption appears to be a predisposing factor in the onset of several civilisation diseases, particularly red meat and its products. One way to reduce diet-related diseases is to guide consumers towards consciously purchasing healthier foods by including a nutrition declaration on product labels, such as by using a “front-of-pack” (FOP) labelling system. This study aimed to determine the Nutri-Score classes for processed meat products, distinguish products that are potentially better for consumers, and determine whether the refined algorithm significantly contributed to a change in product classification. An analysis of the labels of 1700 products available on the Polish market indicated that most processed meat products qualified as class D and E. Comparing the refined Nutri-Score calculation algorithm with the original algorithm resulted in a slight change in product allocation. Poultry products were ranked more favourably than red meat products. The most significant change in product allocation (by 35.2%) was achieved by reducing salt content by 30% and fat content by 10%. Among the processed meat products, some are more highly ranked and are hence considered better from a nutritional perspective than others in that group.

## 1. Introduction

Meat and meat products are an important part of the human diet [1]. In the U.S. and other developed countries, meat provides humans with 15–17% of their daily energy needs and 30–40% of their daily protein needs while also accounting for 20% of their daily fat intake; however, these values vary considerably in different parts of the world [2,3,4].

Red meat, mainly processed red meat, appears to be a predisposing factor for many civilisation diseases. Numerous reports have shown that increased consumption of red meat and processed meat is associated with an increased risk of type 2 diabetes [5,6,7,8,9,10], cardiovascular diseases [11], and ischaemic heart disease [6,8]. Consumption of red and/or processed meat also increases the risk of stroke [12,13,14], particularly ischaemic stroke [15]. Furthermore, increased consumption of processed meat has been related to an increased risk of various cancers, including oesophageal squamous cell carcinoma [16,17], gastric cancer [18], cancers of the large intestine, colon, and rectum [19,20,21], pancreatic cancer [22], and breast cancer [23,24,25,26]. Studies have also identified a relationship between red and processed meat consumption and any-cause mortality [11,27,28]. High consumption of red and processed meat is known to increase the risk of total, cardiovascular, and cancer mortality [29,30]. Some studies have indicated that reducing processed meat consumption below 20 g/day would prevent more than 3% of all deaths [31]. Thus, limiting the consumption of red and processed meat appears to be an essential dietary recommendation for preventing cardiovascular diseases, type 2 diabetes, and various types of cancer.

Poland is characterised by a very high level of processed meat consumption [32], ranking third in the world after Panama and Latin American countries [33]; indeed, in Poland, nearly 17.6% of energy in the diet comes directly from meat products [2]. Therefore, it seems appropriate to encourage consumers to purchase healthier foods consciously by including a nutrition declaration on product labels.

The negative impact of processed meat products on human health could be ameliorated by reformulating products; for example, it might be possible to reduce the levels of sodium [34] or fat. Moreover, some research suggests that the consumption of white meat, an excellent protein source, may be associated with a reduced risk of stomach cancer [18] and stroke [13] compared to red meat.

Within the European Union, in accordance with Regulation (EU) 1169/2011, food products must be labelled with information on the energy value of the product and its fat, saturated fat, carbohydrate, sugar, protein, and salt content; these data must be presented in a tabular form and be legible to consumers. In addition, food business operators (FBOs) may include additional information on these labels, i.e., the amounts of monounsaturated and polyunsaturated fats, polyols, starch, fibre, vitamins, and minerals [35]. Moreover, in accordance with Article 35 of Regulation (EU) 1169/2011, the energy value and the amounts of nutrients may be repeated in the form of graphics or symbols [35] on the front of the packaging, a location known as “front-of-pack” (FOP).

FOP labelling has also been recommended by the WHO to counter the growing obesity epidemic and the increasing risk of non-communicable diseases arising from dietary sources. It has been reported that easy-to-understand food labelling systems can support nutritional education and help consumers choose healthier products while also influencing FBOs to reformulate their products to be healthier [36,37,38,39,40].

Due to the lack of a global, jointly developed labelling system, both within and outside the European Union, individual countries have adopted different forms of labelling, which are either obligatory or voluntary, depending on the local legislation. Indications can be divided into “nutrient-specific” schemes, which provide information on specific nutrients in a numerical and “colour-coded” form, or “summary indicator” schemes. An example of the former is the “NutrInform Battery” adopted in Italy, which illustrates the suggested daily quantity consumption of energy and nutrients contained in a single portion of food as a percentage in the form of a battery symbol [41]. Another colour-coded FOP scheme has been introduced in the UK; this presents the particular nutritional and energy value contained in a single portion of the product and the percentage of an adult’s reference intake, with colour coding (red, amber, and green) [42].

“Summary indicator” schemes can be divided into those including only “positive” indicators, i.e., labelling can only be applied to foods that meet certain criteria, and “graded” indicators, i.e., labelling can be applied to all products that receive a graded designation depending on the adopted scale [43]. Examples of “graded” indicators include the Health Star Rating (HSR, system of classification of health stars) [40], introduced in 2014 in Australia and New Zealand, and the Nutri-Score introduced in 2017 in France. In the case of the Nutri-Score, the information provided on a label is expressed as a five-point colour scale running from dark green to dark orange. Each colour is additionally assigned a letter from A to E. The product is rated on the Nutri-Score scale according to both negative factors (*N*-components), such as sugars, saturated fat, salt, and energy value provided, and positive factors (*P*-components), such as protein, fibre, fruits, vegetables, legume, and nut content [44]. This classification programme has also been implemented in other European countries, such as Spain, Belgium, the Netherlands, Luxembourg, Germany, and Switzerland [43,45]. However, the Nutri-Score scale has been banned by the Italian and Romanian governments, among others, with it being considered an unfair commercial practice [46,47,48].

The purpose of this study was to determine the distribution of processed meat products in individual Nutri-Score classes, thus distinguishing those that offer potentially better nutritional quality for consumers. It also determines how the distribution of individual products in the Nutri-Score classes was affected by reducing their sodium and saturated fat content and whether the use of a refined algorithm significantly contributed to a change in product classification.

## 2. Materials and Methods

### 2.1. Data Collection

The labels of an assortment of food products were reviewed for information on the nutritional value, ingredients, presence of food additives, and type of meat used in production (animal species). All products were classified as processed meat, including meat preparations and meat products, according to the definitions presented in Regulation 853/2004 [49]. The labels were gathered from 75 grocery stores representing five retail chains with the largest market share based on total revenues for 2018 [50], viz. Biedronka (JERONIMO MARTINS POLAND), Lidl (FRF Beteiligungs GmbH), Eurocash, a group that brings together stores such as Lewiatan, Groszek, and Delikatesy Centrum, and the Auchan. Finally, as the Tesco chain has since withdrawn from the Polish market, it was replaced by the Kaufland chain. Sampling was carried out in stores representing each of the above-mentioned retail chains in 11 cities with a population of more than 250,000 and six smaller cities [51], provided that the chains had a branch in the selected city. The labels were collected over a few months, from October 2020 to March 2021.

A total of 12,333 labels from meat preparations or meat products were taken for the study. The analysis identified 1967 unique processed meat products; of these 1700 assortments provided complete data, and only these were used for further analysis, accounting for 86.43% of the total sample. As the labels are not required to carry information on fibre content, an element of the algorithm, a request was sent to the relevant FBO to provide the necessary data. Data on fibre content was thus obtained for 1326 products, i.e., 78% of the analysed assortments. If no response was obtained, the fibre values were assigned based on the average value of the other products in the same assortment group.

### 2.2. Classification of Products

**Figure 1:**
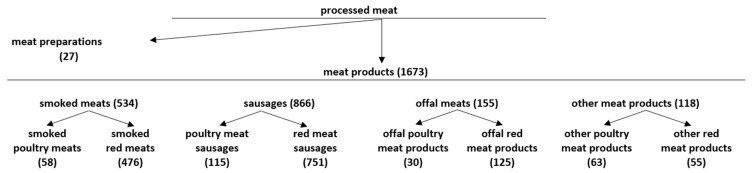
Technological and assortment groups of processed meat products analysed during the research.

Processed meat was divided into two categories, viz. meat products and meat preparations according to the definitions given in Regulation (EC) 853/2004 [49]. The meat products were then divided into smoked meats, sausages, offal meats, and other meats, according to the criteria in the Polish Standard [52]. Based on the information contained in Regulation (EC) 853/2004, the meat was divided into red meat, i.e., of domestic ungulates, referred to in Section I of Annex III of the regulation, and white meat, i.e., of poultry and lagomorphs by Section II of Annex III of the regulation [49]. Due to the wide variety of products in these groups, they were divided further into subgroups according to meat species composition. A detailed breakdown of the groups is presented in Figure 1.

### 2.3. Nutri-Score Calculations

Each product was analysed based on individual nutrient content per 100 g of food using the proposed algorithm [53]. The main algorithm of the Nutri-Score was modified by the Scientific Committee of the Nutri-Score (ScC) in 2022, inter alia, with regard to how the protein content in processed meat products is evaluated. Therefore, two calculations are presented below, i.e., one for the algorithm before the change (hereafter referred to as the *original algorithm*) and another after the change (hereafter referred to as the *refined algorithm*). The processed meat products were scored as follows: for *N*-components, i.e., for energy (0–10 points), sugars (0–10 points), saturated fatty acids (0–10 points), and sodium calculated from the salt content (0–10 points), and for *P*-components, i.e., for the content of fruits, vegetables, pulses, nuts, and rapeseed walnut and olive oils (%) (0–5 points), fibre (0–5 points), and protein (0–5 points in the original algorithm; 0–5 points for white meat or 0–2 points for red meat and its products according to the refined algorithm). The method of assigning points is shown in more detail in Table 1.

Since FBOs are not required to list the sodium content on their labels, the value was calculated based on the amount of salt included on the label i.e., the salt equivalent content [53,54]. The sodium content, (mg), can be calculated by dividing the amount of salt on the label (in g) by 2.5 and multiplying it by 1000.

Then, according to the rules of the algorithm, if the sum of the points awarded for the *N*-component was less than 11, the sum of the *P*-component points was subtracted from the figure obtained. The same calculation method was used where the sum of *N*-component points was greater than or equal to 11, but the number of points awarded for fruits, vegetables, pulses, nuts, and rapeseed walnuts and olive oils (%) was 5. However, if the total *N*-component value was greater than or equal to 11 but the number of points allocated for fruits, vegetables, pulses, nuts, and rapeseed walnut and olive oils (%) was less than 5 points, the points allocated for fruits, vegetables, pulses, nuts, rapeseed walnut and olive oils (%), and fibre were subtracted from the total *N*-component values.

Following this, based on the score, the product was then assigned to the appropriate class from A to E. Class A (dark green)included products that scored −1 point or less; class B (light green) included products that scored from 0 to 2 points; class C (yellow) included products that scored from 3 to 10 points; class D (orange) included products that scored from 11 to 18 points; and class E (dark orange) included products that scored 19 points and above. The results of calculations made using the original algorithm were then compared with those of the refined algorithm.

Reformulation scenarios were also carried out, which included a 30% reduction in salt (sodium) and a 10% reduction in saturated fats both alone and in combination. The reformulation scenarios were based, in the case of salt, on the WHO’s recommendation of a 30% reduction in salt (sodium) intake [55] and a 10% reduction in saturated fats.

### 2.4. Statistical Analyses

The distribution of the tested variables was verified with the Shapiro–Wilk test, which indicated that none were normally distributed. Therefore, further data analysis was performed using Kruskal–Wallis analysis of variance and logistic regression in SPSS software v29.0 (Armonk, NY, USA).

The Kruskal–Wallis analysis of variance was used to assess the differences in the following nutritional characteristics between different processed meat products: total score, energy (KJ), energy score, sugar content, sugar score, saturated fat acid content, SFA score, sodium content, sodium score, protein content, protein score, fibre content, and fibre score. In total, nine groups of products were included in the analysis: meat preparations, smoked poultry meats, smoked red meats, poultry meat sausages, red meat sausages, offal poultry meat products, offal red meat products, other poultry meat products, and other red meat products.

The effect of salt content on the presence of flavour enhancers in meat products was determined using logistic regression. In the model, the dependent variable was the presence of the flavour enhancer in the product (marked as 1) or the lack of the flavour enhancer in the product (marked as 0). The salt content was used as a covariate. The model was validated with the presence of correctly classified cases and the ROC curve.

## 3. Results

### 3.1. Distribution of the Nutri-Score Classes within Different Food Groups

**Figure 2:**
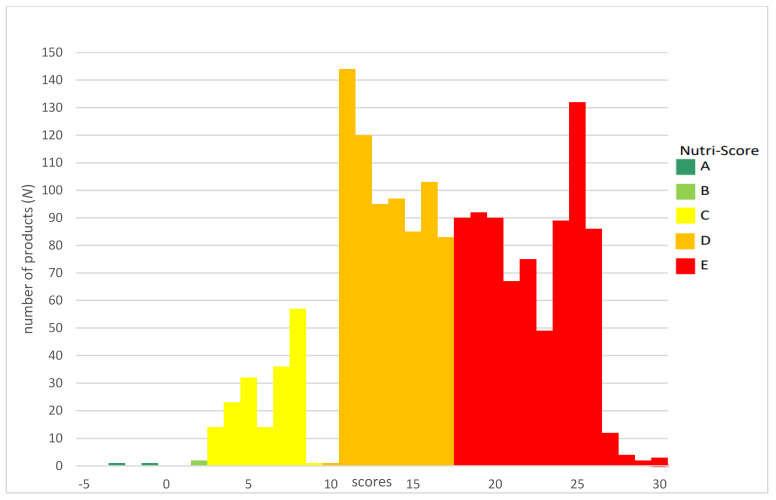
Nutri-Score distribution among yjr analysed processed meat products from the Polish market.

Of the 1967 products obtained from the stores on the days of the survey, 1700 products had sufficient data to perform Nutri-Score calculations and were included in the analysis. Of the studied groups, meat products were much more common on the Polish market, accounting for more than 98.4% of all processed meat products, compared to meat preparations accounting for less than 1.6%. The most widely represented group within the category of meat products was sausages (*N* = 866, 51.76%), followed by smoked meats (*N* = 534, 31.92%), offal meats (*N* = 155, 9.26%) and formed meats (*N* = 118, 7.05%). Based on the refined algorithm, the largest number of processed meat products fell into the Nutri-Score class D (*N* = 817, 48.06%), with slightly fewer products in class E (*N* = 701, 41.24%) and significantly fewer products in class C (*N* = 178, 10.47%). Only four products, accounting for less than 0.25%, fell into classes A and B (Figure 2).

### 3.2. Comparison of Original and Refined Algorithms

The comparison of the original and refined algorithms indicated no difference in the number of products categorised as D and E between the two. In addition, only a virtually imperceptible (*N* = 5, 0.29%) reduction in the number of products categorised as B was noted in favour of C. The distribution of the Nutri-Score classes based on the original and refined algorithms according to food group and subgroup is shown in Table 2.

**Figure 3:**
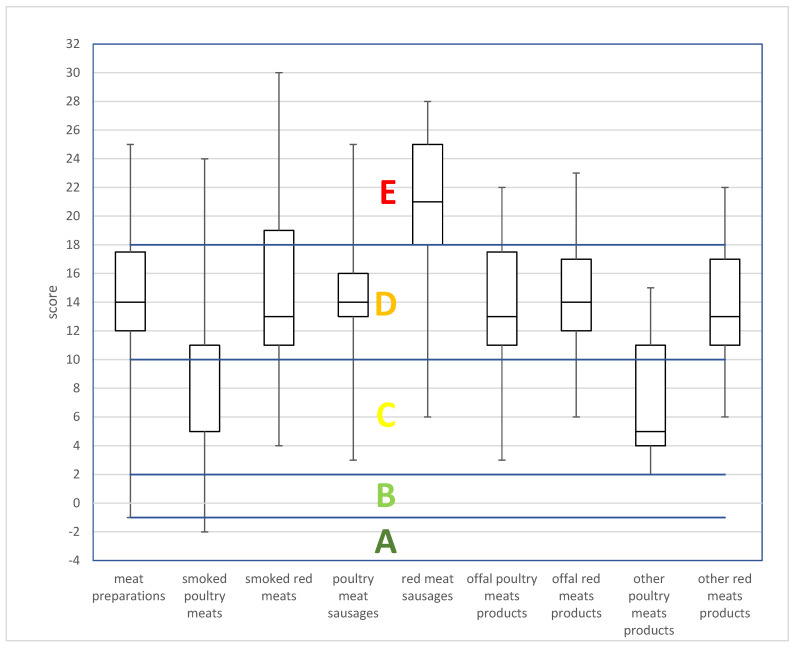
Overall distribution of products within the different food groups according to the refined algorithm. Horizontal lines represent the cut-offs of the five-category Nutri-Score. The box boundaries indicate the 25th percentile (lower boundary) and 75th percentile (top boundary), and the line within the box marks the median. Whiskers (error bars) at the bottom and top of the box indicate the minimum and maximum values.

The distribution of products between classes based on the refined algorithm is illustrated in Figure 3. It also indicates the minimum and maximum point value awarded, the median value, and the dominant Nutri-Score class for each group of processed meat products.

### 3.3. Comparison of White and Red Meat Products

**Figure 4:**
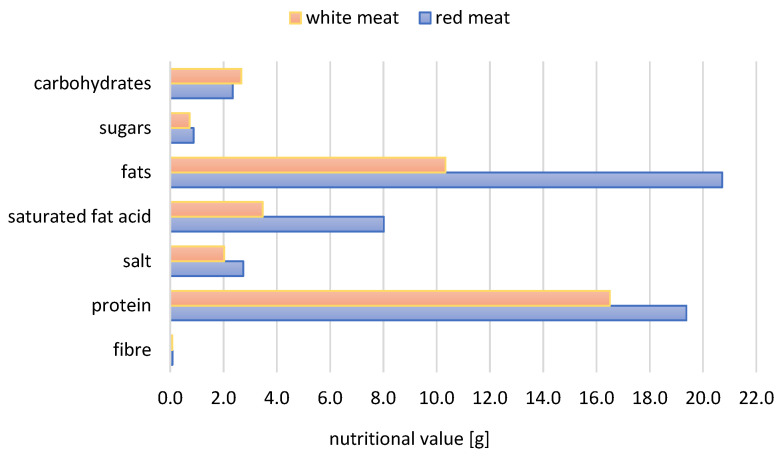
Average nutritional value in grams per 100 g of processed meat products according to their type.

Significant differences in Nutri-Score classification were found between white and red meat products, resulting from their different nutritional value. The energy density of white processed meat products is over one-third lower than that of red processed meat products, with it being on average about 706 kJ. Processed meat products made from red meat are characterised by higher levels of most nutrients per 100 g of the product, especially fat and SFAs, compared to white meat. A detailed comparison of the mean nutritional characteristics of those products is presented in Figure 4.

**Figure 5:**
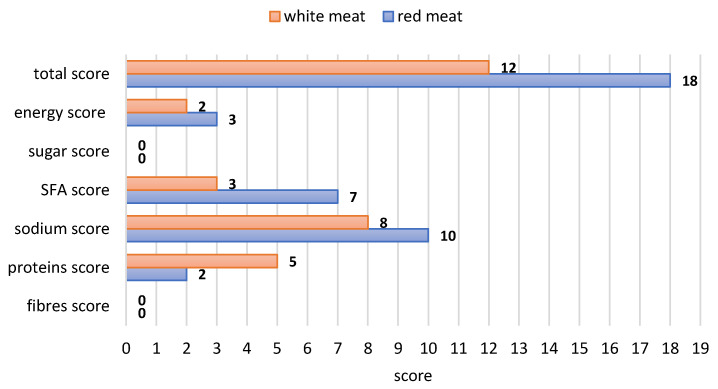
The average number of points awarded in particular categories during the assessment of nutritional value according to the Nutri-Score algorithm.

**Figure 6:**
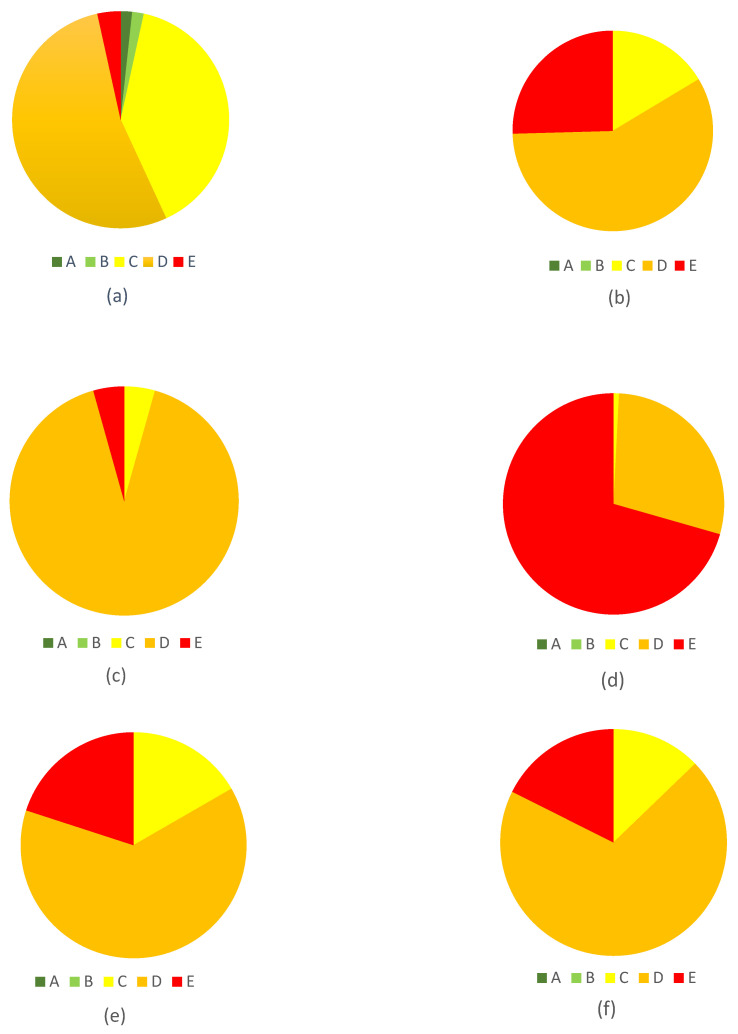

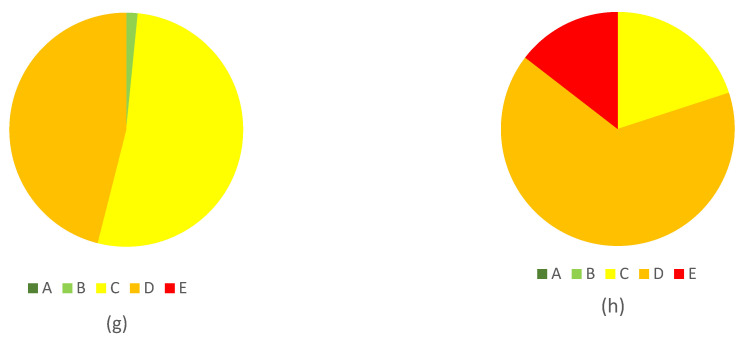
Nutri-Score distributions in the four food groups of smoked meats, sausages, offal meats, and other meat products: (**a**) smoked poultry meats, (**b**) smoked red meats, (**c**) poultry meat sausages, (**d**) red meat sausages, (**e**) offal poultry meat products, (**f**) offal red meat products, (**g**) other poultry meat products, and (**h**) other red meat products.

The above differences are also reflected in the number of points awarded to particular products using the Nutri-Score algorithm (Figure 5); they indicated a more favourable classification for white meat products. The differences in algorithm components for the four main studied food groups are illustrated in Figure 6.

### 3.4. The Formulation Change Scenarios

The median scores for the *N*-components and *P*-components in each group of processed meat products were estimated based on the identified nutritional values of the processed meat products. Among the *N*-components, sodium as a component of salt received the highest score. The median point value for salt for all studied products was nine; the second and third places among the *N*-components went to saturated fatty acids, with six points, and energy density, with two points. None of the products received points for fruits, vegetables, pulses, nuts, and rapeseed walnut or olive oils, as these did not account for 40% of their composition. The median scores for fibre and sugars were zero (the means were 0.04 and 0.02, respectively) and, therefore, did not significantly affect the result of the calculations according to the Nutri-Score algorithm. The detailed data are included in Table 3 and Table 4.

**Figure 7:**
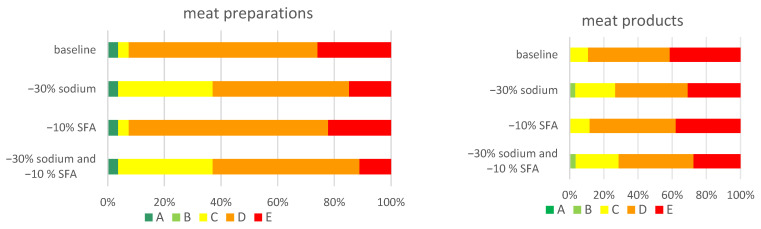
Modifications in product allocations between the Nutri-Score classes depending on the selected formulation change scenario.

As a result of the formulation change, significant modifications in product allocations between Nutri-Score classes were observed. A 30% reduction in salt (sodium) resulted in a class change for 505 products, while a 10% reduction in saturated fatty acids resulted in a class change for 76 products. When the two were combined, it resulted in a classification change for 598 products. The modification of the allocation of meat preparations and meat products is presented in Figure 7. In both food categories, the use of the sodium reduction scenario results in a visible increase in the number of products classified in Nutri-Score class C while reducing the number of assortments classified in class E. Detailed data for the other food groups and subgroups are included in Table 5.

### 3.5. Salt Content and the Presence of Flavour Enhancers in Products

**Figure 8:**
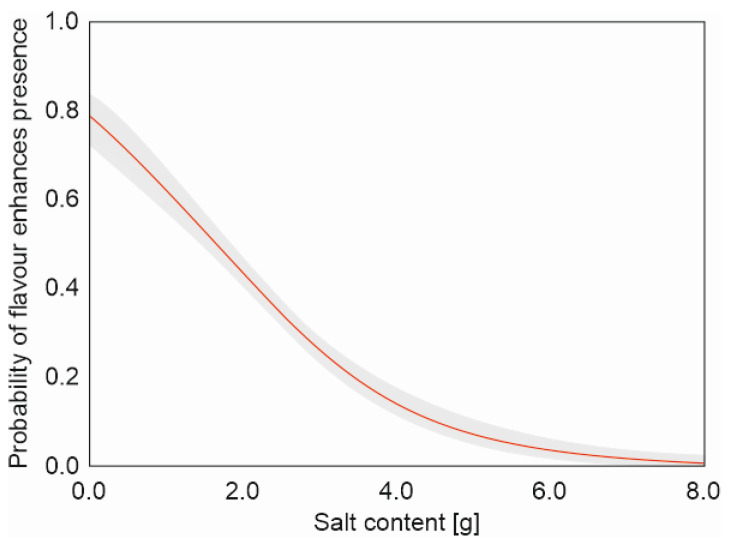
Probability (and CIs) of flavour enhancers being present against salt content in the meat products.

Salt content significantly explained the presence of flavour enhancers in the products. The probability of flavour enhancers being present in the products decreased as the salt content increased (*Χ*^2^ = 168.4, *df* = 1, *p* < 0.001) (Figure 8). However, the model was not well fitted because the percentage of correctly classified cases was only 63, and although the ROC was statistically significant, the AUC value was not high (AUC = 0.649).

## 4. Discussion

The introduction of front-of-pack (FOP) labelling has two primary goals: the first is to help consumers make healthier food choices and the second is to encourage manufacturers to reformulate their current products or create new products that are considered healthier [56].

This study is the first to comprehensively describe the distribution of processed meat products according to Nutri-Score class. The results indicate that 89.3% of processed meat products on the Polish market are classified as class D or E. They should therefore be classified as being for limited consumption, which is in accordance with Polish dietary guidelines recommending, inter alia, more limited consumption of red meat and processed meat products [57,58]. Similar to a study conducted by Dréano-Trécant et al., which analysed products from eight European countries, including Poland, the present study found that processed meat could be classified into all five Nutri-Score classes [59]. Even so, the dominant class in the case of five countries, viz. Finland, Norway, Portugal, Sweden, and Poland, was class D, which is consistent with the results obtained in the present study. In the case of France and Switzerland, the dominant class was class E, and in Slovakia, it was class A [59]. A study performed on the German market found the total percentage of processed meat products categorised as D and E to be 95.8%, i.e., slightly higher than the present study, with the dominant class being E [60].

Our findings indicate that the use of the refined algorithm did not alter the allocation for products allocated to classes D and E. Although reducing the maximum number of points for the protein *P*-component, from five to two points [54], resulted in an increase in the mean number of points awarded to products in class C, it did not affect their movement to class D. However, a class change was noted for five products originally classified as B, which were classified into class C by the refined algorithm. Thus, our study indicates that the change in the algorithm does not cause any significant changes in the distribution of products between classes in the Polish market. Nevertheless, research conducted in the Belgian, French, German, and Dutch markets found that the use of the improved Nutri-Score algorithm resulted in a significant reallocation of processed meat products between classes. An increase of 16 percentage points in the E-class was noted in France, and 10 in the Netherlands, while in Germany, the percentage of products classified into the E-class more than doubled from 8 to 17% [54].

Among the products belonging to the same food group, both the original and refined Nutri-Score algorithms ranked products made from poultry meat more favourably than red meat. As such, the change in scoring to promote poultry meat over red meat did not affect this. In the case of poultry sausages, nearly 91.5% of the products were categorised as D, while in the case of sausages made from red meat, nearly 71% were categorised as E. This may support research that suggests that increasing the consumption of white meat over red meat may be associated with a reduced risk of stomach cancer [18] and stroke [13].

Research in many countries shows that the placement of “Front-of-Pack” labels increases the ability of consumers to evaluate products regarding their nutritional quality and thus make healthier food choices [61,62,63]. It has been proposed that systems which rank products from more favourable to more negative are more understandable to consumers than those using only positive or negative information on the labels [64]. Furthermore, *interpretative* systems, particularly the Nutri-Score, have been found to offer consumers more help in ranking the overall nutritional quality of food products in numerous European countries. In Belgium, France, Germany, Spain, the United Kingdom, Denmark, Bulgaria, the Netherlands, Switzerland, Portugal, Greece, and Poland, the Nutri-Score emerged as the most efficient approach to informing consumers about the nutritional quality of food products [62,65,66,67,68,69,70,71,72]. This could be attributed to the ease with which consumers interpret labels incorporating colour-coding [68,73] as opposed to nutrient-specific systems; the latter heavily depend on numerical information, demanding a cognitive workload that may impede comprehension and utilisation in purchasing scenarios [68].

Furthermore, Engell et al. report that FOP labels can contribute to a decrease in mortality associated with diet-related non-communicable diseases, with Nutri-Score proving to be the most effective among them. It is estimated that around 3.4% of all cases of deaths from diet-related non-communicable diseases could be prevented through the implementation of a Nutri-Score FOP label [74] and that the Nutri-Score FOP labels promote healthier dietary decisions among individuals dealing with chronic cardiometabolic diseases [75].

In the authors’ opinion, processed meat products and their groups exhibit a wide variability in nutritional quality, and it is not easy for an average consumer to see the difference between them. As such, the use of a five-point scale for evaluating processed meat products could allow consumers to effectively distinguish the nutritional quality of different types of processed meats within the same group; in such cases, proper product labelling would allow them to make more conscious decisions. However, Nutri-Score is an interpretative system, not an information system; while it may help consumers choose a better product from a specific product group, it provides limited information for people with specific dietary needs [76].

The use of interpretative labels on the front of packaging, such as Nutri-Score, often faces criticism. For example, identical scores can be obtained by products with diverse nutritional characteristics, and the score does not provide information about specific nutrients like sugars, salt, and saturated fats, which may be important to particular consumer groups; as such, products with the same rating may have different effects on health depending on individual characteristics [77]. Therefore, according to Carruba et al., Nutri-Score FOP labels do little to help individuals identify foods more or less suited to their specific needs [76]. In addition, the algorithm used for the Nutri-Score labelling system involves assessing the content of selected ingredients and energy per 100 g of the product without considering the size of the food portion that is usually consumed. Consequently, there is a risk that better-rated products may be generally consumed in larger quantities, especially when they come in large packages; such greater consumption can have a more significant impact on the overall nutritional quality of the diet compared to other foods with less favourable Nutri-Score ratings but may be consumed in much smaller amounts [76,78].

Julia et al. demonstrated that a nutritional label based on FSA score could help consumers make healthier food choices and potentially play a role in long-term obesity prevention [79]. Also, Egnell et al. report that consumers with lower dietary indices based on nutrient profiling systems (NPS), used to classify foods according to their nutritional value, had a lower body mass index (BMI) gain over time and were much less likely to become overweight [80]. In contrast, Carruba et al. propose that the Nutri-Score scale does not help maintain a normal BMI and does not reduce the likelihood of becoming overweight or developing obesity, mainly because this rating refers to 100 g of the product and not to the portion size, making it challenging to monitor energy intake and control body weight [76].

Nevertheless, implementing FOP labelling has encouraged manufacturers to reformulate their current products or create new products considered healthier, resulting in significant public health benefits [81,82]. After being introduced in New Zealand by the National Heart Foundation of New Zealand (NHF), the FOP Tick programme has allowed consumers to identify healthier product options in relation to heart disease within the same food category; this has led to FBOs excluding 33 tons of salt from breakfast cereals, breads, and margarine products in one year by reformulating these products [82,83]. A similar scenario was observed in Australia [84]. In addition, in order to meet its requirements, the products bearing the logo of the FOP Tick programme are characterised by lower energy, trans-fatty acids (TFAs), saturated fatty acids (SFAs), and sodium contents [85], and the inclusion of the programme’s logo became part of the marketing strategy of many food companies [85].

Similar results were obtained following the introduction of the FOP Health Check programme in Canada and the Choices logo in the Netherlands, which prompted food manufacturers in the country to reformulate existing products and develop new ones with healthier ingredients [56,86]. As a result, total fat, SFAs, TFAs, sodium, and sugar intake was reduced, and fibre intake increased [56]. Moreover, an analysis performed in New Zealand, two years after introducing the FOP Health Star Rating, the successor of the FOP Tick programme, found some foods to have been reformulated as healthier products [87].

Our present findings indicate that the processed meat products received the most points from in the *N*-component group due to their sodium content as a component of salt. This is in line with WHO data indicating that most people consume too much salt [34], with Poland demonstrating the highest salt intake among nine studied European countries [88]. These data, together with estimates that 20–30% of salt in the diet comes from meat and processed meat products [89,90] emphasises the importance of reducing salt consumption from meat products. High salt intake may be associated with many adverse health effects, such as an increased risk of hypertension [89,91], which in turn results in an increased risk of stroke and cardiovascular disease [92]; it is also associated with higher cardiovascular mortality [93] and all-cause premature mortality [94,95], as well as an increased risk of stomach cancer and kidney disease and a higher risk of becoming overweight and developing obesity [91]. However, it is important to remember that sodium is an essential nutrient, and too little sodium also negatively affects the human body by increasing the levels of renin and aldosterone [96]. Indeed, the lowest risk of cardiovascular events and death from such events is observed in populations with a medium sodium intake [96,97,98].

All WHO member states pledged in 2013 to implement programmes to reduce salt intake from food and that the amount of salt consumed in food should be reduced by 30% by 2025 [55]. Hendriksen et al. estimated that a 30% reduction in dietary salt intake in Poland would reduce the incidence of stroke by 13.5%, ischaemic heart disease by 8.9%, and deaths from cardiovascular disease by 5% [88]. In the present study, a simulated 30% reduction in the amount of salt used in processed meat products (Table 5) resulted in a 29.71% modification of product allocation between categories: a marked decrease in the number of products assigned to class E and an increase in the number of products assigned to class B. As such, it can be speculated that considering only the nutritional values, reducing the amount of salt used in processed meat products will make these products healthier for consumers.

However, reduced-salt meat products often have a different texture and flavour to those produced conventionally; such changes may foster consumer aversion to them, and limit the effectiveness of the salt reduction programmes [99]. In addition to influencing the taste of food [98,99], salt plays many other functions in food products, such as extending shelf life and preserving food by reducing water activity [88,89,100,101,102]; it can also act as a binding agent between meat and fat and promote the dissolution of microfibrillar proteins to maintain the proper texture of products [88,89]. Hence, the primary challenge facing the meat industry is to reduce salt concentrations while maintaining the sensory acceptability, cost, with salt being cheaper than substitutes [98], and safety of processed meat products [103,104].

Our study found that lower-salt products were more likely to contain flavour enhancers than salt-rich products. Often, the development of reduced-salt products involves greater use of flavour enhancers and agents to mask an undesirable taste [89,105,106]. Such substitutes include other chloride salts, such as KCl [107,108,109,110], CaCl_2_, and MgCl_2_ [111,112], non-chloride salts, such as lactate and diacetate [113], or flavour enhancers, such as arginine, lysine, sodium inosinate, sodium guanylate, taurine, glycine, yeast extracts, and monosodium glutamate [105,114,115,116].

Our research showed that processed meat products also received a high score in the *N*-component group, particularly regarding saturated fatty acid (SFA) content. High fat intake has been linked to the onset of many lifestyle diseases, and excessive consumption of SFAs is a factor in cardiovascular disease [117,118]. It has been found that removing SFAs can result in healthier processed meat products and that choosing low-fat food may lower the risk of colorectal cancer (CRC) associated with consuming processed meat [119].

However, it is important to remember, that in addition to providing energy, fats strongly influence the consistency, taste and appearance of a product, in addition to its characteristic structure and flavour [120]. Therefore, limiting the SFA content in a product may involve the need to use fat substitutes to maintain its sensory attractiveness [120,121]. Most importantly, these substitutes will also affect the energy content of the product and, thus, its overall Nutri-Score rating.

Research indicates that the Nutri-Score is well perceived and understood by consumers and has performed well in studies comparing the nutritional quality of products. Food labelling on the front of the package is an effective way to in promote consumer awareness and can help consumers make beneficial choices [63,75,122,123,124,125]. However, it should be noted that the Nutri-Score system has some limitations: the rating only applies to 100 g of the product and not a typical food portion, and due to its simplified nature, the algorithm does not address other aspects of the products, such as certain food additives or mechanically separated meat, or their mineral, vitamin, or specific fatty acid content [78]. Furthermore, the results show that the presence of FOP labels may reduce the attention consumers pay to the nutritional information on food packaging, suggesting possible over-reliance on the information presented on the front. This is often the case with interpretative labels offering a summary of the overall healthfulness of the product, like Nutri-Score [126].

Our study has some limitations. Due to our desire to obtain a comprehensive overview of the Polish market and include as many products as possible, our analyses were based on data obtained from processed meat product labels. Therefore, our calculations were based on the results of chemical tests of products carried out by FBOs and not on our chemical analyses. Consequently, the study was restricted to products that provided complete information on their labels. Moreover, because labels are not required to carry information on fibre content (an element of the algorithm), these data had to be obtained directly from the FBOs; in some cases where the exact data were not available, average values were assigned. Furthermore, it was not possible to calculate a precise energy score in the 10% SFA reduction scenario as it was unclear whether the SFAs had been replaced with a fat substitute and which type. As such, the product may have been underestimated or overestimated with regard to its final score.

To effectively support the harmonisation of FOP labelling in European countries, there is clearly a need for more comprehensive research considering other food groups on the European and Polish markets. These studies should focus on determining the effectiveness of the refined Nutri-Score algorithm in helping consumers identify the nutritional quality of a food item. Moreover, as the Nutri-Score algorithm is based on the analysis of ingredients in 100 g of a product, and not a standard consumed portion, it seems advisable to determine whether it has any real impact on limiting the consumption of ingredients considered potentially undesirable.

We believe that our study results can become a starting point for conducting such research, and to extend this to encompass products not included in the Nutri-Score algorithm, such as food additives and minerals or vitamins. It can also examine the relationship between their occurrence and concentration in the Nutri-Score class.

## 5. Conclusions

In summary, almost 90% of the meat products available on the Polish market were found to fall into Nutri-Score classes D and E, with poultry meat products being classified more favourably than red meat products. The investigated products were awarded the highest negative scores for sodium content, followed by SFA content. A 30% reduction in salt content significantly altered the classification for 505 products, while a 10% reduction in SFA content resulted in a class change for 76 products. The simultaneous application of both scenarios resulted in the reclassification of 598 products. In addition, products with a lower salt content were more likely to contain flavour enhancers, most likely to improve the sensory value.

Our research shows that among processed meat, it is possible to distinguish assortments that have been ranked higher than others and are considered better for the consumer from a nutritional value point of view. Applying the refined algorithm did not significantly modify the allocation of processed meat products on the Polish market; this finding casts doubt on the value of the introduced changes regarding the Nutri-Score classification of processed meat.

Hence, there is a need for more information regarding the effectiveness by which Nutri-Score can be used to distinguish nutritional quality in countries applying the refined algorithm. The findings could play an important role in introducing a harmonised system of FOP labelling in European Union countries.

## Figures and Tables

**Table 1:** Method of awarding points to a food product according to its content.

Points	*N*-Component				*P*-Component			
	Energy Density (kJ/100 g)	Sugar (g/100 g)	Saturated Fatty Acids (g/100 g)	Sodium (mg/100 g)	Fruits, Vegetables, Pulses, Nuts, and Rapeseed Walnut and Olive Oils (%)	Fibre (g/100 g)	Protein (g/100 g)	
							All Meat in the Original Algorithm and White Meat in the Refined Algorithm	Red Meat in the Refined Algorithm
0	≤335	≤4.5	≤1	≤90	≤40	≤0.9	≤1.6	≤2.4
1	>335	>4.5	>1	>90	>40	>0.9	>1.6	>2.4
2	>670	>9	>2	>180	>60	>1.9	>3.2	>4.8
3	>1005	>13.5	>3	>270	-	>2.8	>4.8	-
4	>1340	>18	>4	>360	-	>3.7	>6.4	-
5	>1675	>22.5	>5	>450	>80	>4.7	>8.0	-
6	>2010	>27	>6	>540	-	-	-	-
7	>2345	>31	>7	>630	-	-	-	-
8	>2680	>36	>8	>720	-	-	-	-
9	>3015	>40	>9	>810	-	-	-	-
10	>3350	>45	>10	>900	-	-	-	-

**Table 2:** Distribution of processed meat products, divided by group and Nutri-Score class, based on the original and refined algorithms.

	Class	Algorithm	Meat Preparations	Smoked Poultry Meats	Smoked Red Meats	Poultry Meat Sausages	Red Meat Sausages	Offal Poultry Meat Products	Offal Red Meat Products	Other Poultry Meat Products	Other Red Meat Products
Number of products (N)	A	original	1	1	0	0	0	0	0	0	0
		refined	1	1	0	0	0	0	0	0	0
	B	original	0	1	5	0	0	0	0	1	0
		refined	0	1	0	0	0	0	0	1	0
	C	original	1	23	73	5	6	5	16	33	11
		refined	1	23	78	5	6	5	16	33	11
	D	original	18	31	277	105	215	19	87	29	36
		refined	18	31	277	105	215	19	87	29	36
	E	original	7	2	121	5	530	6	22	0	8
		refined	7	2	121	5	530	6	22	0	8
Mean score (SD)		original	14.33	8.93	14.18	14.12	20.85	13.43	14.21	7.75	13.24
		refined	14.44	8.93	14.67	14.12	20.88	13.43	14.54	7.75	13.84

**Table 3:** Nutritional characteristics of processed meat products according to their type. The values are given as the median and interquartile range: x (y, z) where “x” is the median value, “y” indicates the 25th percentile, and “z” indicates the 75th percentile.

	Meat Preparations		Smoked Poultry Meats		Smoked Red Meats		Poultry Meat Sausages		Red Meat Sausages		Offal Poultry Meat Products		Offal Red Meat Products		Other Poultry Meat Products		Other Red Meat Products	
N	27		58		476		115		751		30		125		63		55	
total score	14.0	(12.0, 17.5)	11.0	(5.0, 11.0)	13.0	(11.0, 19.0)	14.0	(13.0, 16.0)	21.0	(18.0, 25.0)	13.0	(11.0, 18.0)	14.0	(12.0, 17.0)	5.0	(4.0, 11.0)	13.0	(11.0, 17.0)
KJ	806	(430, 990)	477	(436, 625)	718	(501, 1041)	810	(718, 900)	1256	(1019, 1778)	923	(762, 1089)	956	(779, 1201)	464	(412, 632)	714	(436, 887)
energy score	2.0	(1.0, 2.0)	1.0	(1.0, 1.0)	2.0	(1.0, 3.0)	2.0	(2.0, 2.0)	3.0	(3.0, 5.0)	3.0	(2.0, 3.0)	2.0	(2.0, 3.0)	1.0	(1.0, 1.0)	2.0	(1.0, 2.0)
sugars	0.5	(0.5, 0.7)	0.6	(0.5, 0.7)	0.5	(0.5, 0.8)	0.7	(0.5, 1.1)	0.7	(0.5, 1.0)	1.0	(0.6, 2.0)	0.7	(0.5, 1.0)	0.6	(0.5, 0.8)	0.7	(0.5, 1.0)
sugar score	0.0	(0.0, 0.0)	0.0	(0.0, 0.0)	0.0	(0.0, 0.0)	0.0	(0.0, 0.0)	0.0	(0.0, 0.0)	0.0	(0.0, 0.0)	0.0	(0.0, 0.0)	0.0	(0.0, 0.0)	0.0	(0.0, 0.0)
SFA	6.0	(1.8, 8.6)	1.0	(0.6, 2.5)	3.4	(1.4, 6.5)	4.6	(3.5, 5.2)	10.0	(7.4, 14.0)	4.7	(3.4, 7.9)	6.0	(4.2, 9.0)	1.2	(0.7, 2.6)	4.3	(1.4, 6.4)
SFAs score	5.0	(1.0, 8.0)	0.0	(0.0, 2.0)	3.0	(1.0, 6.0)	4.0	(3.0, 5.0)	9.0	(7.0, 10.0)	4.0	(3.0, 7.0)	5.0	(4.0, 8.0)	1.0	(0.0, 2.0)	4.0	(1.0, 6.0)
sodium	880	(680, 940)	840	(800, 950)	960	(800, 1440)	800	(760, 880)	1040	(800, 1400)	680	(600, 750)	680	(600, 760)	800	(720, 840)	880	(760, 980)
sodium score	9.0	(7.0, 10.0)	9.0	(8.0, 10.0)	10.0	(8.0, 10.0)	8.0	(8.0, 9.0)	10.0	(8.0, 10.0)	7.0	(6.0, 8.0)	7.0	(6.0, 8.0)	8.0	(7.0, 9.0)	9.0	(8.0, 10.0)
protein	15.6	(14.7, 17.0)	20.0	(16.0, 22.0)	20.0	(16.0, 25.0)	15.0	(13.0, 17.7)	20.0	(15.0, 25.0)	12.0	(9.1, 15.0)	13.0	(9.4, 15.0)	17.0	(14.0, 19.0)	14.0	(11.6, 16.5)
protein score	2.0	(2.0, 2.0)	5.0	(5.0, 5.0)	2.0	(2.0, 2.0)	5.0	(5.0, 5.0)	2.0	(2.0, 2.0)	5.0	(5.0, 5.0)	2.0	(2.0, 2.0)	5.0	(5.0, 5.0)	2.0	(2.0, 2.0)
fibre	0.5	(0.5, 0.5)	0.0	(0.0, 0.0)	0.0	(0.0, 0.0)	0.0	(0.0, 0.1)	0.0	(0.0, 0.0)	0.2	(0.1, 0.4)	0.2	(0.1, 0.4)	0.0	(0.0, 0.0)	0.0	(0, 0)
fibre score	0.0	(0.0, 0.0)	0.0	(0.0, 0.0)	0.0	(0.0, 0.0)	0.0	(0.0, 0.0)	0.0	(0.0, 0.0)	0.0	(0.0, 0.0)	0.0	(0.0, 0.0)	0.0	(0.0, 0.0)	0.0	(0.0, 0.0)

**Table 4:** Nutritional characteristics of meat products (cat. 8.3) according to their type, divided into particular Nutri-Score classes. The values are given as the median and interquartile range: x (y, z), where “x” is the median value, “y” indicates the 25th percentile, and “z” indicates the 75th percentile.

	**Smoked Poultry Meats**										**Smoked Red Meats**							
	**A**		**B**		**C**		**D**		**E**		**A**	**B**	**C**		**D**		**E**	
N	1		1		22		30		2		0	0	78		277		121	
total score	−3.0		2.0		5.0	(4.0, 5.0)	11.0	(11.0, 12.0)	24.0	(24.0, 24.0)			8.0	(7.0, 8.0)	13.0	(12.0, 15.0)	22.0	(20.0, 24.0)
KJ	346		481		438	(404, 455)	544	(482, 658)	1474	1460, 1487)			478	(435, 541)	650	(498, 875)	1266	(1145, 1539)
energy score	1.0		1.0		1.0	(1.0,1.0)	1.0	(1.0, 1.5)	4.0	(4.0, 4.0)			1.0	(1.0, 1.0)	1.0	(1.0, 2.0)	3.0	(3.0, 4.0)
sugars	0.1		0.0		0.6	(0.5, 0.7)	0.5	(0.5, 0.9)	0.7	(0.6, 0.7)			0.6	(0.5, 0.9)	0.5	(0.5, 0.9)	0.5	(0.5, 0.6)
sugar score	0.0		0.0		0.0	(0.0, 0.0)	0.0	(0.0, 0.0)	0.0	(0.0, 0.0)			0.0	(0.0, 0.0)	0.0	(0.0, 0.0)	0.0	(0.0, 0.0)
SFAs	1.1		0.5		0.5	(0.3, 0.8)	2.1	(0.9, 3.0)	11.0	(11.0, 11.0)			1.1	(0.8, 1.5)	2.6	(1.5, 4.4)	11.0	(8.3, 13.0)
SFA score	1.0		0.0		0.0	(0, 0)	2.0	(0.0, 2.0)	10.0	(10.0, 10.0)			1.0	(0.0, 1.0)	2.0	(1.0, 4.0)	10.0	(8.0, 10.0)
sodium	80		600		800	(780, 880)	920	(800, 1000)	1580	(1530, 1630)			760	(720, 800)	1000	(880, 1840)	1040	(880, 1840)
sodium score	0.0		6.0		8.0	(8.0, 9.0)	10.0	(8.0, 10.0)	10.0	(10.0, 10.0)			8.0	(7.0,8.0)	10.0	(9.0, 10.0)	10.0	(9.0, 10.0)
protein	13.0		25.0		20.0	(18.5, 22.0)	17.0	(15.0, 22.0)	20.0	(20.0, 20.0)			20.0	(18.0, 22.8)	21.0	(16.9, 26.0)	17.0	(13.0, 26.0)
proteins score	5.0		5.0		5.0	(5.0, 5.0)	5.0	(5.0, 5.0)	5.0	(5.0, 5.0)			2.0	(2.0, 2.0)	2.0	(2.0, 2.0)	2.0	(2.0, 2.0)
fibre	0.0		0.0		0.0	(0.0, 0.0)	0.0	(0.0, 0.0)	0.0	(0.0, 0.0)			0.0	(0.0, 0.0)	0.0	(0.0, 0.0)	0.0	(0.0, 0.0)
fibre score	0.0		0.0		0.0	(0.0, 0.0)	0.0	(0.0, 0.0)	0.0	(0.0, 0.0)			0.0	(0.0, 0.0)	0.0	(0.0, 0.0)	0.0	(0.0, 0.0)
	**Poultry Meat Sausages**										**Red Meat Sausages**							
	**A**		**B**		**C**		**D**		**E**		**A**	**B**	**C**		**D**		**E**	
N	0		0		5		105		5		0	0	6		215		530	
total score					5.0	(4.0, 5.0)	14.0	(13.0, 15.0)	20.0	(19.0, 25.0)			8.0	(7.0, 8.0)	16.0	(14.0, 18.0)	24.0	(21.0, 25.0)
KJ					567	(554, 591)	810	(725, 886)	1152	(1108, 1708)			667	(492, 624)	944	(816, 1012)	1503	(1200, 1894)
energy score					1.0	(1.0, 1.0)	2.0	(2.0, 2.0)	3.0	(3.0, 3.0)			1.0	(1.0, 1.0)	2.0	(2.0, 3.0)	4.0	(3.0, 5.0)
sugars					0.5	(0.5, 0.6)	0.5	(0.4, 0.7)	0.7	(0.7, 1.1)			0.8	(0.7, 1.0)	0.5	(0.5, 0.7)	0.8	(0.5, 1.4)
sugar score					0.0	(0.0, 0.0)	0.0	(0.0, 0.0)	0.0	(0.0, 0.0)			0.0	(0.0, 0.0)	0.0	(0.0, 0.0)	0.0	(0.0, 0.0)
SFAs					1.5	(0.8, 1.5)	4.6	(3.7, 5.2)	8.6	(7.7, 11.0)			1.4	(1.3, 1.6)	6.0	(4.1, 7.3)	12.0	(9.6, 15.0)
SFA score					1.0	(0.0, 1.0)	4.0	(3.0, 5.0)	8.0	(7.0, 10.0)			1.0	(1.0, 1.0)	5.0	(4.0, 7.0)	10.0	(9.0, 10.0)
sodium					720	(680, 800)	800	(760, 840)	1080	(920, 1120)			706	(603, 800)	840	(720, 980)	1240	(920, 1520)
sodium score					7.0	(7.0, 8.0)	8.0	(8.0, 9.0)	10.0	(10.0, 10.0)			7.0	(6.0, 8.0)	9.0	(7.0, 10.0)	10.0	(10.0, 10.0)
protein					23.5	(18.0, 26.0)	15.0	(13.0, 17.0)	14.0	(13.0,15.0)			20.0	(19.3, 20.8)	17.0	(14.0, 23.0)	22.0	(15.0, 25.0)
protein score					5.0	(5.0, 5.0)	5.0	(5.0, 5.0)	5.0	(5.0, 5.0)			2.0	(2.0, 2.0)	2.0	(2.0, 2.0)	2.0	(2.0, 2.0)
fibre					0.0	(0.0, 0.0)	0.0	(0.0, 0.1)	0.0	(0.0, 0.1)			0.0	(0.0, 0.0)	0.0	(0.0, 0.1)	0.0	(0.0, 0.1)
fibre score					0.0	(0.0, 0.0)	0.0	(0.0, 0.0)	0.0	(0.0, 0.0)			0.0	(0.0, 0.0)	0.0	(0.0, 0.0)	0.0	(0.0, 0.0)
	**Offal Poultry Meat Products**										**Offal Red Meat Products**							
	**A**		**B**		**C**		**D**		**E**		**A**	**B**	**C**		**D**		**E**	
N	0		0		5		19		6		0	0	16		87		22	
total score					5.0	(4.0, 5.0)	14.0	(13.0, 15.0)	20.0	(19.0, 25.0)			7.0	(7.0, 8.0)	14.0	(13.0, 16.0)	21.0	(20.0, 22.0)
KJ					588	(514, 760)	920	(769, 994)	1218	(1215, 1318)			687	(604, 740)	914	(786, 1109)	1439	(1293, 1587)
energy score					1.0	(1.0, 2.0)	2.0	(2.0, 3.0)	3.0	(3.0, 4.0)			1.0	(1.0, 1.0)	2.0	(2.0, 4.0)	4.0	(3.0, 4.0)
sugars					0.7	(0.6, 0.8)	1.6	(0.8, 2.1)	0.7	(0.4, 1.4)			0.7	(0.5, 1.2)	0.7	(0.5, 1.0)	0.8	(0.6, 1.0)
sugar score					0.0	(0.0, 0.0)	0.0	(0.0, 0.0)	0.0	(0.0, 0.0)			0.0	(0.0, 0.0)	0.0	(0.0, 0.0)	0.0	(0.0, 0.0)
SFAs					2.9	(1.9, 3.0)	4.5	(3.9, 6.0)	8.7	(8.5, 9.0)			2.8	(1.6, 3.3)	5.9	(4.5, 8.1)	12.9	(10.5, 14.0)
SFA score					2.0	(1.0, 2.0)	4.0	(3.0, 6.0)	10.0	(10.0, 10.0)			2.0	(1.0, 3.0)	5.0	(4.0, 8.0)	10.0	(10.0, 10.0)
sodium					600	(600, 720)	680	(620, 760)	600	(600, 720)			580	(430, 650)	680	(600, 732)	760	(680, 790)
sodium score					6.0	(6.0, 7.0)	7.0	(7.0, 8.0)	6.0	(6.0, 8.0)			6.0	(4.0, 7.0)	7.0	(6.0, 8.0)	8.0	(7.0, 8.0)
protein					15.0	(12.0, 15.0)	14.0	(11.5, 18.0)	8.7	(8.5, 9.0)			10.8	(8.8, 15.0)	13.1	(10.0, 15.0)	12.0	(8.7, 14.0)
protein score					5.0	(5.0, 5.0)	5.0	(5.0, 5.0)	5.0	(5.0, 5.0)			2.0	(2.0, 2.0)	2.0	(2.0, 2.0)	2.0	(2.0, 2.0)
fibre					0.1	(0.1, 0.2)	0.4	(0.2, 0.4)	0.1	(0.1, 0.1)			0.2	(0.1, 0.2)	0.2	(0.1, 0.5)	0.2	(0.1, 2.1)
fibre score					0.0	(0.0, 0.0)	0.0	(0.0, 0.0)	0.0	(0.0, 0.0)			0.0	(0.0, 0.0)	0.0	(0.0, 0.0)	0.0	(0.0, 0.2)
	**Other Poultry Meat Products**										**Other Red Meat Products**							
	**A**		**B**		**C**		**D**		**E**		**A**	**B**	**C**		**D**		**E**	
N	0		1		33		29				0	0	11		36		8	
total score			2.0		4.0	(4.0, 5.0)	11.0	(11.0, 13.0)					8.0	(7.0, 8.0)	15.0	(12.0, 17.0)	22.0	(20.0, 22.0)
KJ			311		436	(407, 467)	585	(481, 721)					429	(371, 472)	744	(455, 849)	1458	(1193, 1635)
energy score			0.0		1.0	(1.0, 1.0)	1.0	(1.0, 2.0)					1.0	(1.0, 1.0)	2.0	(1.0, 2.0)	4.0	(3.0, 4.0)
sugars			0.8		0.7	(0.5, 0.9)	0.6	(0.4, 0.7)					0.7	(0.7, 0.8)	0.6	(0.5, 0.9)	0.9	(0.4, 1.5)
sugar score			0.0		0.0	(0.0, 0.0)	0.0	(0.0, 0.0)					0.0	(0.0, 0.0)	0.0	(0.0, 0.0)	0.0	(0.0, 0.0)
SFAs			1.4		0.8	(0.6, 1.2)	2.6	(1.2, 3.6)					1.3	(1.0, 1.8)	4.5	(1.5, 6.0)	8.7	(7.7, 10.5)
SFA score			1.0		0.0	(0.0, 1.0)	2.0	(1.0, 3.0)					1.0	(0.0, 1.0)	4.0	(1.0, 5.0)	8.0	(7.0, 9.0)
sodium			600		800	(720, 800)	840	(720, 1000)					800	(740, 820)	880	(800, 1000)	1020	(758, 1760)
sodium score			6.0		8.0	(7.0, 8.0)	9.0	(7.0, 10.0)					8.0	(8.0, 9.0)	9.0	(8.0, 10.0)	10.0	(8.0, 10.0)
protein			8.2		18.0	(16.0, 19.0)	17.0	(13.0, 18.0)					15.0	(14.0, 16.5)	13.5	(11.0, 15.3)	12.0	(11.0, 39.5)
protein score			5.0		5.0	(5.0, 5.0)	5.0	(5.0, 5.0)					2.0	(2.0, 2.0)	2.0	(2.0, 2.0)	2.0	(2.0, 2.0)
fibre			0.5		0.0	(0.0, 0.0)	0.0	(0.0, 0.1)					0.0	(0.0, 0.0)	0.0	(0.0, 0.1)	0.0	(0.0, 0.1)
fibre score			0.0		0.0	(0.0, 0.0)	0.0	(0.0, 0.0)					0.0	(0.0, 0.0)	0.0	(0.0, 0.0)	0.0	(0.0, 0.0)

**Table 5:** Distribution across Nutri-Score classes and change from the baseline distribution according to product reformulation. The values are given as “N”—the number of products and the “%”—the percentage of products assigned to a given Nutri-Score class in relation to all products in this food group.

Assortments Group				Nutritional Information Class									
				A		B		C		D		E	
				N	%	N	%	N	%	N	%	N	%
Baseline	meat preparations			1	3.70	0	0.00	1	3.70	18	66.67	7	25.93
	meat products			1	0.06	2	0.12	177	10.58	799	47.76	694	41.48
		smoked meats		1	0.19	1	0.19	101	18.91	308	57.68	123	23.03
			smoked poultry meats	1	1.72	1	1.72	23	39.66	31	53.45	2	3.45
			smoked red meats	0	0.00	0	0.00	78	16.39	277	58.19	121	25.42
		sausages		0	0.00	0	0.00	11	1.27	320	36.95	535	61.78
			poultry meat sausages	0	0.00	0	0.00	5	4.35	105	91.30	5	4.35
			red meat sausages	0	0.00	0	0.00	6	0.80	215	28.63	530	70.57
		offal meats		0	0.00	0	0.00	21	13.55	106	68.39	28	18.06
			offal poultry meat products	0	0.00	0	0.00	5	16.67	19	63.33	6	20.00
			offal red meat products	0	0.00	0	0.00	16	12.80	87	69.60	22	17.60
		other meat products		0	0.00	1	0.85	44	37.29	65	55.08	8	6.78
			other poultry meat products	0	0.00	1	1.59	33	52.38	29	46.03	0	0.00
			other red meat products	0	0.00	0	0.00	11	20.00	36	65.45	8	14.55
Minus 30% sodium	meat preparations			1	3.70	0	0.00	9	33.33	13	48.15	4	14.81
	meat products			1	0.06	53	3.17	392	23.43	710	42.44	517	30.90
		smoked meats		1	0.19	21	3.93	230	43.07	178	33.33	104	19.48
			smoked poultry meats	1	1.72	20	34.48	29	50.00	6	10.34	2	3.45
			smoked red meats	0	0.00	1	0.21	201	42.23	172	36.13	102	21.43
		sausages		0	0.00	3	0.35	57	6.58	409	47.23	397	45.84
			poultry meat sausages	0	0.00	3	2.61	25	21.74	85	73.91	2	1.74
			red meat sausages	0	0.00	0	0.00	32	4.26	324	43.14	395	52.60
		offal meats		0	0.00	2	1.29	51	32.90	91	58.71	11	7.10
			offal poultry meat products	0	0.00	2	6.67	12	40.00	15	50.00	1	3.33
			offal red meat products	0	0.00	0	0.00	39	31.20	76	60.80	10	8.00
		other meat products		0	0.00	27	22.88	54	45.76	32	27.12	5	4.24
			other poultry meat products	0	0.00	27	42.86	27	42.86	9	14.29	0	0.00
			other red meat products	0	0.00	0	0.00	27	49.09	23	41.82	5	9.09
Minus 10% SFAs	meat preparations			1	3.70	0	0.00	1	3.70	19	70.37	6	22.22
	meat products			1	0.06	2	0.12	192	11.48	844	50.45	634	37.90
		smoked meats		1	0.19	1	0.19	107	20.04	315	58.99	110	20.60
			smoked poultry meats	1	1.72	1	1.72	25	43.10	29	50.00	2	3.45
			smoked red meats	0	0.00	0	0.00	82	17.23	286	60.08	108	22.69
		sausages		0	0.00	0	0.00	13	1.50	357	41.22	496	57.27
			poultry meat sausages	0	0.00	0	0.00	7	6.09	104	90.43	4	3.48
			red meat sausages	0	0.00	0	0.00	6	0.80	253	33.69	492	65.51
		offal meats		0	0.00	0	0.00	23	14.84	111	71.61	21	13.55
			offal poultry meat products	0	0.00	0	0.00	6	20.00	22	73.33	2	6.67
			offal red meat products	0	0.00	0	0.00	17	13.60	89	71.20	19	15.20
		other meat products		0	0.00	1	0.85	49	41.53	61	51.69	7	5.93
			other poultry meat products	0	0.00	1	1.59	38	60.32	24	38.10	0	0.00
			other red meat products	0	0.00	0	0.00	11	20.00	37	67.27	7	12.73
Minus 30% sodium and 10% SFAs	meat preparations			1	3.70	0	0.00	9	33.33	14	51.85	3	11.11
	meat products			1	0.06	57	3.41	421	25.16	734	43.87	460	27.50
		smoked meats		1	0.19	21	3.93	239	44.76	191	35.77	82	15.36
			smoked poultry meats	1	1.72	20	34.48	31	53.45	4	6.90	2	3.45
			smoked red meats	0	0.00	1	0.21	208	43.70	187	39.29	80	16.81
		sausages		0	0.00	3	0.35	72	8.31	427	49.31	364	42.03
			poultry meat sausages	0	0.00	3	2.61	33	28.70	77	66.96	2	1.74
			red meat sausages	0	0.00	0	0.00	39	5.19	350	46.60	362	48.20
		offal meats		0	0.00	3	1.94	57	36.77	86	55.48	9	5.81
			offal poultry meat products	0	0.00	3	10.00	12	40.00	14	46.67	1	3.33
			offal red meat products	0	0.00	0	0.00	45	36.00	72	57.60	8	6.40
		other meat products		0	0.00	30	25.42	53	44.92	30	25.42	5	4.24
			other poultry meat products	0	0.00	30	47.62	25	39.68	8	12.70	0	0.00
			other red meat products	0	0.00	0	0.00	28	50.91	22	40.00	5	9.09

## Data Availability

The data presented in this study are available from the corresponding author upon request.
